# First report of morphological and molecular characterization of Moroccan populations of *Globodera pallida*

**DOI:** 10.21307/jofnem-2021-007

**Published:** 2021-02-25

**Authors:** A. Hajjaji, R. Ait Mhand, N. Rhallabi, F. Mellouki

**Affiliations:** Research Unit Microbiology, Hygiene and Biomolecules, Laboratory of Virology, Microbiology, Quality and Biotechnology/Ecotoxicology and Biodiversity, University Hassan II of Casablanca, FSTM, Casablanca, Morocco; Plant Quarantine Laboratory, Bouznika, Morocco

**Keywords:** *Globodera pallida*, *Globodera rostochiensis*, Potato, Cyst nematodes, Molecular-identification, RFLP, Morocco

## Abstract

Potato cyst nematodes (PCNs) are the most important potato pest causing major crop losses across the world with a quarantine status in many countries. In Morocco, several potato crops are infected with PCNs and the monitoring of potato production as well as the control of import and export of potato seeds are currently carried out by morphological methods. The present work was aimed to use molecular and morphometric methods for identifying and differentiating PCN species in Morocco for the first time. The morphological identification of PCN species from collected soil samples were carried out using the shape of the cysts, the length of the stylet, the number of cuticular ridges, and the Granek’s ratio. The J2 had a slightly shorter body length, the number of cuticular ridges was 9 and the Granek’s ratio averaged 2.2. The morphobiometric analysis revealed proximity of the Moroccan population to *G. pallida* species. PCNs sampled from contaminated fields were analyzed molecularly using PCR. DNA amplification was performed using the multiplex PCR method and PCR-RFLP from the ITS region of the total genomic DNA compared to multiplex PCR-specific DNA sequences. All confirmed the presence *G. pallida* in all samples of the Moroccan PCN populations.

Potato cyst nematodes (PCNs) are soil-borne plant pests which have great economic importance to potato production that are internationally recognized quarantine pests (EPPO, 2004). In Morocco, they are regulated by sanitary rules for imported plants or plant products (Decree of the Minister of Agriculture and Agrarian Reform of Morocco, 1986).

The plant parasitic nematode *Globodera pallida* (Stone, 1973) Behrens is the greatest threat to potato crops and other Solanaceous hosts (Sullivan, 2007). It originates from the Andean mountains of South America from where it was transported to other regions in the world by potato seed (EPPO, 2004; Marks and Brodie, 1998). *G. pallida* has been reported in most countries of western Latin America, eastern North America, Central America, and Europe (EPPO, 2020). In Africa, the presence of *G. pallida* has been reported in Algeria (Mezerket et al., 2018), Tunisia (Hlaoua et al., 2008), and Kenya (Mburu et al., 2018). *G. pallida* does not produce rapid and obvious symptoms on the potato especially for the first months of field infestation. Then it damages the roots and reduces the production yields. Damages can appear to be symptoms of water stress or mineral deficiencies.

Morphological identification of *Globodera* species is an initial means of differentiation of *Globodera* species that are morphologically and morphometrically closely related that ultimately requires molecular confirmation. Molecular diagnosis by the polymerase chain reaction (PCR) test is the most reliable method for the detection of PCN species (Powers, 2004). PCR with specific primers used in single or multiplex reactions and PCR-RFLP (Restriction Fragment Length Polymorphisms) are among the tests developed to identify *Globodera* species (Fullaondo et al., 1999; Blok et al., 1998; Bulman and Marshall, 1997; Vejl et al., 2002).

The present study was conducted in different regions known to be the main potato production areas in Morocco. The main objective was to carry out a morphological identification with more reliable molecular methods to identify and differentiate Moroccan populations of PCNs.

## Material and methods

### Nematode collection

**Figure 1:**
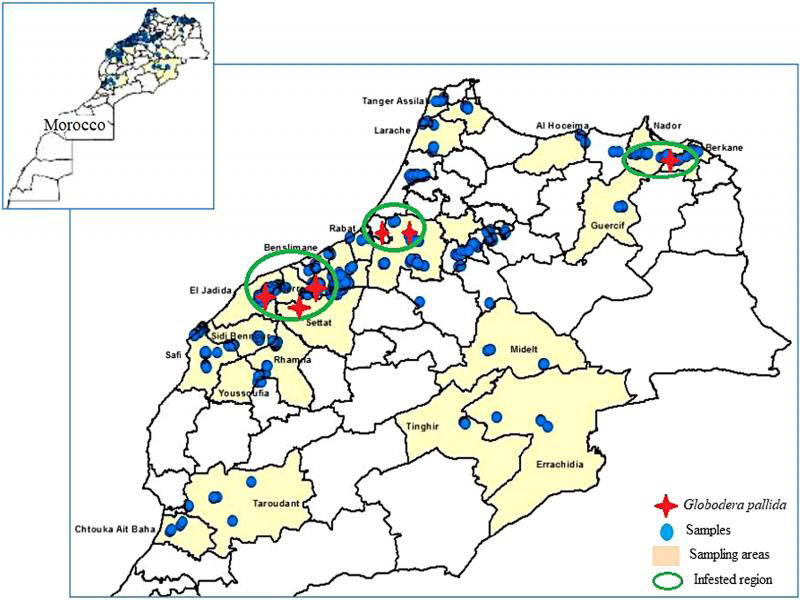
Sampled areas and regions infested with *Globodera pallida* in Morocco.

Soil samples were collected in 2019 to 2020 just before the harvest of potato in different potato-producing regions of Morocco: central western (Casablanca-Rabat), Eastern (Nador-Berkane), northern (Larache-Tangier), central (middle Atlas), south-eastern (Midelt-Errachidia), and (Agadir-Taroudant) locations of the country (Fig. 1). A total of 1,500 soil samples were collected according to the sampling protocol described by the European Plant Protection Directive (69/465/EEC-2007/33/EC) in a rectangular grid covering the entire field, with a minimum width of 5 meters and a maximum length of 20 meters between sampling points. Samples were then transported to the laboratory for analysis. The cysts were extracted by flotation and sieving then, they were visually sorted by stereomicroscope using Fenwick’s apparatus (Fenwick, 1940). The apparatus was filled to the brim with water then dried soil was sprayed through the upper 1 to 2 mm mesh sieve and then into a funnel running down into the body of the apparatus. The cysts float and are dragged by overflow into the recovery collar, under which a 250 µm sieve was placed. The water supply was maintained until the sample was exhausted and clear water was overflowed, then collected cysts were air-dried, placed in 0.5 ml tubes and stored at 4°C until use.

### Morphological identification

In total, 50 *Globodera* cysts per region were prepared for a morphobiometric study on the basis of several characters especially color, size, and Granek’s ratio (the distance between the anus and the vulva divided by the diameter of the vulvar pelvis) and number of cuticular ridges between fenestra and anus for cysts, stylet length, tail length, length of hyaline terminal part of tail. Stylet knob shape of the second-stage juveniles J2 was also used for PCNs identification (EPPO, 2017 PM 7/40). The vulval cones are mounted in a 2% glycerin-agar medium as described by Correia and Abrantes (1997) and the collected larvae are killed under a low flame and then transferred to a drop of immersion oil or a drop of glycerol. Observations and measurements were made using an Olympus BX43 microscope. This operation was conducted with three repetitions and average values were calculated and compared to the corresponding reference data for *Globodera* species (EPPO, 2013).

### DNA extraction

DNA isolation was achieved as described by Ibrahim et al. (2001). Two cyst samples per region were placed in an Eppendorf tube (one cyst by tube) containing 3 × 2.8 mm steel beads and 8 × 1 mm zirconium beads with a 100 µl solution of lysis buffer (Tris HCl 10 mM pH = 8.0; EDTA 1 mM; Nonidet P40 1%; Proteinase K 100 μg/ml final buffer. After that, the cysts were crushed with a Vibro Mixer homogenizer BeadBlaster^TM^ 24, firstly for 40 sec at maximum frequency and incubated at 65°C for 1 hr and then at 95°C for 10 min after removal of the beads (Subbotin et al., 1999). The total genomic DNA suspension was centrifuged at 13,000 rpm for 1 min and was quantified and its purity assessed using Thermo Scientific™ NanoDrop spectrophotometers and then stored at −20°C for later use.

### PCR amplification

The multiplex PCR mixture consisted of 25 μl which contained: Taq DNA polymerase buffer 1X, primers 0.64 µM, MgCl2: 2 mM, dNTPs: 0.25 mM, Taq DNA polymerase (BIOLINE): 0.6 U/reaction, DNA: 20 to 30 ng/µl and Ultra-pure water Qsp 25 μl. The primers used in this study were ITS5, PITSp4, and PITSr3 (Bulman and Marshall, 1997; Skantar et al., 2007; White et al., 1990) and were purchased from BIONEER (Table 1).

**Table 1.** Morphological and morphometric characteristics of Moroccan population of cysts and second-stage juveniles compared to the standard *Globodera pallida* (EPPO, 2004).

Species	Shape of knob	J2 stylet length (µm)	Number of cuticula ridges	Granek’s ratio
*G. rostochiensis*a	Rounded	(21.8)	16-31 (>14)	1.3-9.5 (>3)
*G. pallida* ^a^	Pointed	22-24 (23.8)	8-20 (<14)	1.2-3.5 (<3)
Moroccan population *G. pallida*	Pointed	22.6	9	2.2

**Notes:** ^a^Standard measurement OEPP/EPPO (2004) OEPP/EPPO Bulletin 34: 155-16.

PCR was carried out in an Applied Biosystems ^TM^ ProFlex Thermal Cycler. The cycling program consisted of an initial denaturation step of 2 min at 94°C followed by 35 cycles of denaturation of 30 sec at 94°C, 30 sec at 60°C, 30 sec at 72°C, and final elongation step of 7 min at 72°C. Once the DNA has been amplified, 5 µl of the PCR product was separated electrophoretically using 2% agarose gel in a 1% TAE buffer, stained with SYBR® Safe DNA Gel stain 10,000X, then migrated for 40 min at 100 mA and photographed under UV light. DNA fragment sizes were determined by comparing with the 100 bp DNA marker.

### RFLP-PCR

For this experiment, we used three restriction enzymes (AluI, MboI, and RasI) for DNA samples taken at random from samples of *G. pallida* cyst populations collected from Berkane in the East, Gharb, and Doukkala in the West of Morocco and were identified by morphology. As instructed by the manufacturer (Promega R6281), 1 µg/µl of the PCR product was digested with 10 U/µl restriction enzyme, 10 µg/µl BSA, 10X restriction buffer, and sterile deionized water for a reaction volume of 20 µl. After 4 hr incubation at room temperature at 25°C, the samples were deposited in wells of a 2% agarose gel in TAE 1% buffer stained with SYBR® Safe DNA Gel stain x10,000. The restriction fragments were separated by migration for 40 min at 100 mA and photographed under UV light.

## Results

### Morphological observations

**Figure 2:**
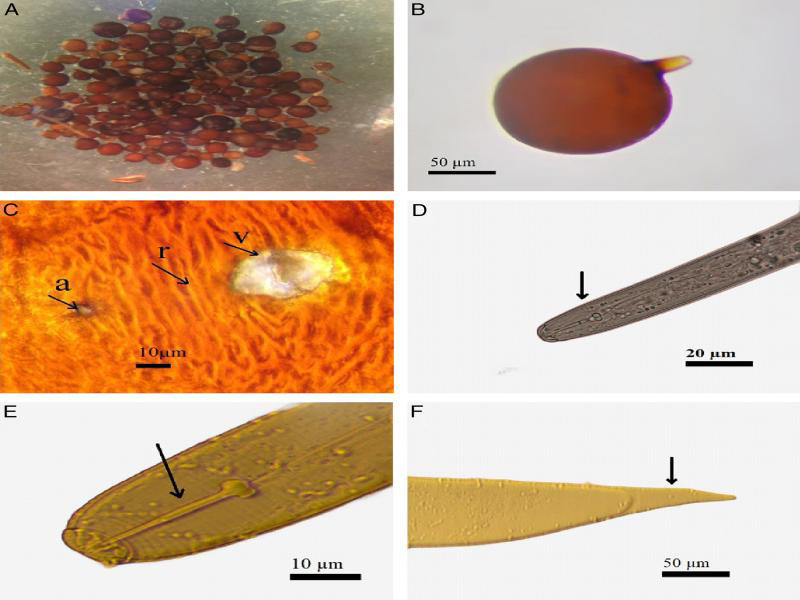
Photomicrographs of morphological characterization of cyst, egg, and second-stage juvenile. A, B: Cyst of *Globodera pallida*, C: Vulva (v), anus (a), and cuticular ridges (r) of cyst, D, E: Stylet knob shape of J2, F: Juvenile tail.

The nematode cysts in all populations studied are spherical without cones, dark brown, and yellow in color (Fig. 2A, B). The cuticular ridges between fenestra and anus are visible (Fig. 2C). The cephalic structure of the juvenile J2 shows a reniform perioral disc laterally flanked by two amphids and bordered by the elliptical rim (Fig. 2D). These structures characteristic of *G. pallida* are consistent with those reported by Stone (1973).

### Morphometric data

Morphometric studies were carried out on 20 cysts and 50 juveniles of PCNs from each potato-producing area found to be infested (Table 2). The measured mean values for cysts were 22.8 ± 2 µm for fenestra diameter, 36.8 ± 1.8 μm for distance from the fenestra to the anus of and 2.1 µm for width. The narrow fenestra structures of the cysts averaged 8.5 µm. The average number of cuticular wrinkles between the anus and the fenestra was 9. The Granek’s ratio averaged 2.2, J2 larvae indicated a stylet length of 22.6 µm (Fig. 2E), tail length between 59 and 67 µm (Fig. 2F) and average length of the hyaline part not exceeding 1.6 times the length of the stylet.

**Table 2.** Primers used in the present study.

Primer name	Nematode	5′-3′ Sequence	Band size	References
ITS5	Forward	GGAAGTAAAAGTCGTAACAAGG	–	White et al. (1990)
PITSp4	*Globodera pallida*	ACAACAGCAATCGTCGAG	265 bp	Bulman and Marshall (1997); Skantar et al. (2007)
PITSr3	*Globodera rostochiensis*	AGCGCAGACATGCCGCAA	434 bp	

All the measured values for cysts and second-stage juveniles were within the range compared to reference data for *G. pallida*.

### Molecular data

**Figure 3:**
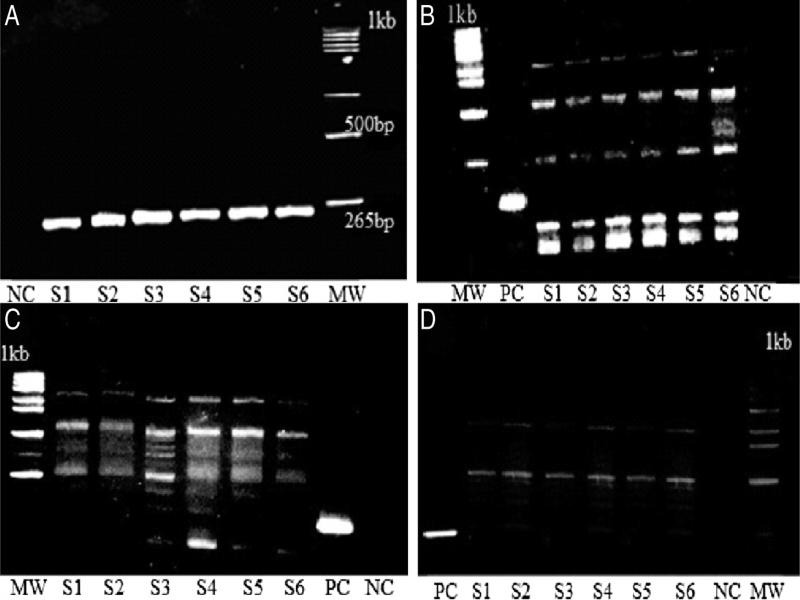
Amplified PCR products from *Globodera spp*. digested by three enzymes AluI, MboI, and RsaI. A: Amplified PCR products, B: AluI, C: RsaI, D: MboI, MW: molecular weight markers (1 kb), NC: negative control, PC: undigested DNA, S1-S2: Eastern region samples, S3-S4: Western region samples (Gharb), S5-S6: Doukkala region samples.

Amplification of the ITS regions of ribosomal DNA for six samples of the three regions by PITSp4 primers produced fragments of approximately 265 bp compared to those of *G. pallida* (Fig. 3A). Moreover, the tested samples targeted with PITSr3primer did not give any amplification and band of 434 bp. These results of multiplex PCR confirmed the results of morphological and morphometric identification, and have proven that all samples tested belong to the species *pallida*.

Digestion of the PCR product with the three restrictions enzymes AluI, MboI, and RsaI resulted in identical fragments for all samples taken from different potato plots. The AluI digestion (Fig. 3B) showed that the samples from the three regions of Morocco are identical and generated five fragments of 100, 200, 500, 700, and 850 bp and we found that the RsaI digestion clearly identified the three populations from the sampling areas being that *Globodera pallida* and revealed three identical fragments of 500, 750, and 850 bp (Fig. 3C). The use of MboI digestion giving two bands of 500 and 700 bp clearly confirmed the identity of the three populations of PCNs even if it revealed poorly visible fragments of less than 100 bp and greater than 900 bp were not included in the analysis (Fig. 3D). These results confirmed that there is no genetic diversity detectable with RFLP technology among species of *Globodera pallida* in all sampled potato-producing regions in Morocco.

## Discussion

In the present study, we used both morphological and molecular methods to characterize the Moroccan populations of the quarantine species of *Globodera*. For morphological determinations, the Granek’s ratio is seen as the most informative morphometric measure currently available for nematodes, especially for separating the two *Globodera* species namely *G. pallida* and *G. rostochiensis* (Fleming and Powers, 1998). Our morphological investigations showed Granek’s ratio values less than 3 and reaching 2.2, the length of the stylet 22.6 µm and the number of cuticular ridges between the anus and the fenestra was 9. All these values were in conformity with reference data described for *G. pallida* (EPPO, 2017 PM 7/40). This is the first time that *G. pallida* is reported from Morocco.

Knowing that *G. rostochiensis* has already been reported from parts of Morocco (Schluter, 1976), we conducted molecular investigations using PCR methods for determination more accurately of *Globodera* populations and avoiding confusion of *G. pallida* with other *Globodera* species. Obtained results allowed discrimination of PCNs and identification of *G. pallida* as the predominate PCN in the investigated potato-producing regions in Morocco. Furthermore, our results revealed a restricted distribution of *G. pallida* in three infested regions namely Berkane in the East, Gharb, and Doukkala in the West. Moreover, the used PCR-RFLP method showed similar RFLP patterns suggesting the presence of the same species, *G. pallida,* in the surveyed areas. Bulman and Marshall (1997), Skantar et al. (2007), White et al. (1990) reported that the use of ITS5, PITSp4, and PITSr3 primers reliably and rapidly characterized *Globodera* PNC species.

Several works have reported the wide prevalence of *Globodera pallida* in Mediterranean countries such as Bello et al. (2005) in northern and southern of Spain, Camacho et al. (2017) in all potato-growing areas in Portugal, and Ambrogioni (1977) in the region of Bari and Napoli in Italy since 1977. In the African continent, *Globodera pallida* has recently been described in three countries, namely six regions in the northern, north-eastern, and central eastern of Tunisia (Hlaoua et al., 2008), on northern of Algeria (Mezerket et al., 2018) and in Kenya (Mburu et al., 2018). Either in Morocco or in other Mediterranean countries, the regions infested by *Globodera pallida* are regions with cold and rainy temperature which could influence on the dissemination of *Globodera pallida* (Kaczmarek et al., 2014).

In the present work, a molecular method confirmed the identification and discrimination of PCN species. The use of species-specific primers in a multiplex PCR reaction for real-time PCR, allowed the identification at the same time of two species in the same sample. Our results demonstrated that PCR methods are suitable for rapid screening of PCN’s samples from infested fields and preventing spread of PCNs.

In conclusion, based on the combined data from morphological and molecular methods, *G. pallida* was identified for the first time in soil samples from the main potato-producing areas of Morocco and was shown to be the most common PCN nematode species in these studied regions. The origin of the presence or introduction of *G. pallida* in Morocco is unknown. The lack of local seed production could have encouraged the introduction and dissemination of this potato pest by import of potato seed from countries infested with this nematode. Thereby, Morocco must take precaution in order to avoid rapid multiplication and dissemination of *Globodera* species to other free regions. Compulsory control measures are to be put in place for an initial period of 6 years. Afterwards control measures may be lifted if no more nematodes are detected. If they are, control measures may be renewed for three years (EU Councel Directive 2007/33/EC, 2007). Other preventive measures must be applied. These include prohibition to plant or store the potatoes or other plants specified, disinfection of farm equipment at the exit of the plot or in contact with contaminated lots, destruction of contaminated batches of potatoes or host plants, elimination of regrowth, and encouragement of farmers to produce and use local seed.
